# Consensus Middle East and North Africa Registry on Inborn Errors of Immunity

**DOI:** 10.1007/s10875-021-01053-z

**Published:** 2021-05-29

**Authors:** Asghar Aghamohammadi, Nima Rezaei, Reza Yazdani, Samaneh Delavari, Necil Kutukculer, Ezgi Topyildiz, Ahmet Ozen, Safa Baris, Elif Karakoc-Aydiner, Sara Sebnem Kilic, Hulya Kose, Nesrin Gulez, Ferah Genel, Ismail Reisli, Kamel Djenouhat, Azzeddine Tahiat, Rachida Boukari, Samir Ladj, Reda Belbouab, Yacine Ferhani, Brahim Belaid, Reda Djidjik, Nadia Kechout, Nabila Attal, Khalissa Saidani, Ridha Barbouche, Aziz Bousfiha, Ali Sobh, Ragheed Rizk, Marwa H. Elnagdy, Mona Al-Ahmed, Salem Al-Tamemi, Gulnara Nasrullayeva, Mehdi Adeli, Maryam Al-Nesf, Amel Hassen, Cybel Mehawej, Carla Irani, Andre Megarbane, Jessica Quinn, MENA-I. E. I. Study Group, Zahra Chavoshzadeh, Seyed Alireza Mahdaviani, Mohammamd Nabavi, Marzieh Tavakol, Nasrin Behniafard, Tooba Momen, Gholamreza Azizi, Mohammad Hassan Bemanian, Saba Arshi, Rasol Molatefi, Roya Sherkat, Afshin Shirkani, Reza Amin, Soheila Aleyasin, Reza Faridhosseini, Farahzad Jabbari-Azad, Hamid Ahanchian, Maryam Khoshkhui, Alireza Shafiei, Arash Kalantari, Iraj Mohammadzadeh, Javad Ghaffari, Taher Cheraghi, Mahboubeh Mansouri, Mehrnaz Mesdaghi, Delara Babaie, Mohammad Hossein Eslamian, Abbas Dabbaghzadeh, Mahmoud Tavassoli, Ramin Ghasemi, Rasoul Nasiri Kalmarzi, Seyed Hamidreza Mortazavi, Sara Kashef, Hossein Esmaeilzadeh, Javad Tafaroji, Abbas Khalili, Fariborz Zandieh, Mahnaz Sadeghi-Shabestari, Sepideh Darougar, Fatemeh Behmanesh, Hedayat Akbari, Mohammadreza Zandkarimi, Farhad Abolnezhadian, Abbas Fayezi, Mehdi Torabizadeh, Mojgan Moghtaderi, Habib Soheili, Akefeh Ahmadiafshar, Behzad Shakerian, Vahid Sajedi, Behrang Taghvaei, Mojgan Safari, Marzieh Heidarzadeh, Babak Ghalebaghi, Seyed Mohammad Fathi, Behzad Darabi, Kian Darabi, Saeed Bazregari, Nasrin Bazargan, Morteza Fallahpour, Alireza Khayatzadeh, Bahram Bashardoust, Homa Sadri, Mohammadali Zamani, Azam Mohsenzadeh, Sarehsadat Ebrahimi, Reza Ghaemi, Fatemeh Zare Mehrjerdi, Samin Sharafian, Seyed Hesamodin Nabavizadeh, Leila Baniadam, Fereshteh Salari, Mahsa Rekabi, Ahmad Vosughimotlagh, Mitra Tafakoridelbari, Ali Pourvali, Arezou Rezaei, Amir Ali Hamidieh, Mansoureh Shariat, Mohammad Gharagozlou, Masoud Movahedi, Nima Parvaneh, Neslihan Edeer Karaca, Guzide Aksu, Sevgi Bilgiç Eltan, Nurhan Kasap, Burcu Kolukisa, Asena Pınar Sefer, Ezgi Yalcin, Roya babayeva, Lydia Lamara Mahammed, Ahmad Al-Khabaz, László Maródi, Vicki Modell, Fred Modell, Waleed Al-Herz, Raif S. Geha, Hassan Abolhassani

**Affiliations:** 1 Research Center for Immunodeficiencies, Pediatrics Center of Excellence, Children’s Medical Center, Tehran University of Medical Sciences, Tehran, Iran; 2 Department of Pediatric Immunology, Ege University Faculty of Medicine, Bornova-İzmir, Turkey; 3 Faculty of Medicine, Pediatric Allergy and Immunology, Marmara University, Istanbul, Turkey; 4 The Isil Berat Barlan Center for Translational Medicine, Istanbul Jeffrey Modell Foundation Diagnostic Center for Primary Immune Deficiencies, Istanbul, Turkey; 5 Uludag University, Medical Faculty, Department of Pediatric Immunology and Rheumatology, Bursa, Turkey; 6 Department of Pediatric Immunology and Allergy, University of Health Sciences Dr. Behçet Uz Children’s Hospital, İzmir, Turkey; 7 Department of Pediatric Immunology and Allergy, Meram Medical Faculty, Necmettin Erbakan University, Konya, Turkey; 8 Laboratory of Immunology, Department of Medical Biology, Rouiba Hospital, Algiers, Algeria; 9 Department of Pediatrics, Mustapha Pacha University Hospital, University of Algiers, Algiers, Algeria; 10 Department of Medical Immunology, University Hospital Center of Beni Messous, University of Algiers, Algiers, Algeria; 11 Department of Immunology, Pasteur Institute of Algeria/Faculty of Medicine, Algiers, Algeria; 12 Institut Pasteur de Tunis, Université Tunis El Manar, Tunis, Tunisia; 13 Laboratory of Clinical Immunology, Inflammation and Allergy, Faculty of Medicine and Pharmacy of Casablanca, King Hassan II University, Casablanca, Morocco; 14 Department of Pediatrics, Mansoura University Children’s Hospital, Faculty of Medicine, Mansoura University, Mansoura, Egypt; 15 Department of Medical Biochemistry and Molecular Biology, Faculty of Medicine, Mansoura University, Mansoura, Egypt; 16 Department of Microbiology, Faculty of Medicine, Kuwait University, Kuwait City, Kuwait; 17 Department of Allergy, Al-Rashid Allergy Center, Kuwait University, Kuwait City, Kuwait; 18 Department of Child Health, Sultan Qaboos University Hospital, Muscat, Oman; 19 Department Immunology Research Laboratory, Azerbaijan Medical University, Baku, Azerbaijan; 20 Allergy and Immunology Division, Pediatrics Department, Sidra Medicine, Doha, Qatar; 21 Allergy and Immunology Section, Department of Medicine, Hamad Medical Corporation, Doha, Qatar; 22 Department of Human Genetics, Gilbert and Rose-Marie Chagoury School of Medicine, Lebanese American University, Byblos, Lebanon; 23 Internal Medicine and Clinical Immunology, Hotel Dieu de France Hospital, Saint Joseph University, Beirut, Lebanon; 24 Jeffrey Modell Foundation (JMF), New York City, NY USA; 25 PID Clinical Unit and Laboratory, Department of Dermatology, Semmelweis University, Budapest, Hungary; 26 St. Giles Laboratory of Human Genetics of Infectious Diseases, Rockefeller University, New York, NY USA; 27 Department of Pediatrics, Faculty of Medicine, Kuwait University, Safat 13110, PO Box 24923, Kuwait City, Kuwait; 28 Allergy and Clinical Immunology Unit, Pediatric Department, Al-Sabah Hospital, Kuwait City, Kuwait; 29 Division of Immunology, Boston Children’s Hospital and Harvard Medical School, 1 Blackfan Circle, Karp, Bldg, 10th Floor, Boston, MA 02115 USA; 30 Division of Clinical Immunology, Department of Laboratory Medicine, Karolinska Institutet, Karolinska University Hospital, 14186 Huddinge, Stockholm Sweden

**Keywords:** Inborn errors of immunity, Primary immunodeficiency, Epidemiology, Burden of disease, Molecular diagnosis

## Abstract

**Background:**

Inborn errors of immunity (IEIs) are a heterogeneous group of genetic defects of immunity, which cause high rates of morbidity and mortality mainly among children due to infectious and non-infectious complications. The IEI burden has been critically underestimated in countries from middle- and low-income regions and the majority of patients with IEI in these regions lack a molecular diagnosis.

**Methods:**

We analyzed the clinical, immunologic, and genetic data of IEI patients from 22 countries in the Middle East and North Africa (MENA) region. The data was collected from national registries and diverse databases such as the Asian Pacific Society for Immunodeficiencies (APSID) registry, African Society for Immunodeficiencies (ASID) registry, Jeffrey Modell Foundation (JMF) registry, J Project centers, and International Consortium on Immune Deficiency (ICID) centers.

**Results:**

We identified 17,120 patients with IEI, among which females represented 39.4%. Parental consanguinity was present in 60.5% of cases and 27.3% of the patients were from families with a confirmed previous family history of IEI. The median age of patients at the onset of disease was 36 months and the median delay in diagnosis was 41 months. The rate of registered IEI patients ranges between 0.02 and 7.58 per 100,000 population, and the lowest rates were in countries with the highest rates of disability-adjusted life years (DALY) and death rates for children. Predominantly antibody deficiencies were the most frequent IEI entities diagnosed in 41.2% of the cohort. Among 5871 patients genetically evaluated, the diagnostic yield was 83% with the majority (65.2%) having autosomal recessive defects. The mortality rate was the highest in patients with non-syndromic combined immunodeficiency (51.7%, median age: 3.5 years) and particularly in patients with mutations in specific genes associated with this phenotype (*RFXANK*, *RAG1*, and *IL2RG*).

**Conclusions:**

This comprehensive registry highlights the importance of a detailed investigation of IEI patients in the MENA region. The high yield of genetic diagnosis of IEI in this region has important implications for prevention, prognosis, treatment, and resource allocation.

**Supplementary Information:**

The online version contains supplementary material available at 10.1007/s10875-021-01053-z.

## Introduction

Inborn errors of immunity (IEIs), formerly known as primary immunodeficiency disorders (PIDs), are a heterogeneous group of diseases that affect the development and/or function of the immune system [1]. More than 450 monogenic diseases (mainly with autosomal recessive inheritance) have been identified in patients with IEI [2–4], resulting in better management and treatment of these patients [5, 6]. The majority of IEI patients are susceptible to recurrent severe infections causing significant morbidity and mortality. However, other non-infectious complications including immune dysregulation, lymphoproliferation, and malignancies have received more attention recently [7]. Although the prevalence of IEI is estimated to be 1:1200 [8], less than 200,000 patients have been diagnosed and reported worldwide [2, 3, 9].

Globally, there are more than 6 million children under the age of 15 years who die annually despite substantial progress in vaccination, adequate nutrition, encouragement of breastfeeding, reduction of air pollution, provision of safe water and food, and adequate sanitation and hygiene. Leading causes of death in children include severe infectious diseases (i.e., pneumonia and diarrhea) which could affect in particular patients with underlying immune defects. Although vaccines are available for some of the deadliest childhood diseases, such as measles, polio, diphtheria, tetanus, and pertussis, pneumonia due to *Haemophilus influenzae* type B and *Streptococcus pneumonia* and diarrhea due to rotavirus, they may not be effective in protecting immunodeficient children from illness and death. The IEI burden has been critically underestimated and thus has not been given priority in policymaking. Governments, policymakers, and private health agencies need to be provided with accurate data in order to develop and enforce measures for early diagnosis of IEI and promote access to appropriate management based on the exact type of the disease.

The Middle East and North Africa (MENA) region comprises a large area with 5.4% of the world’s population. Countries in this region have a high childhood mortality rate and disability-adjusted life years (DALY) [10, 11] (Figure S1). MENA has one of the youngest average population ages (median age of 26.8 years) and one of the world’s most rapidly expanding populations (average annual growth rate of 1.56%). Even though significant improvement has been documented during the last 30 years, MENA epidemiologic indices stay below the overall global average, mainly for children under 1 year of age [11] (Figure S2). Countries in the MENA region have the highest frequency of consanguineous marriage worldwide and are therefore more prone to have autosomal recessive genetic disorders [12]. To evaluate the current status of IEI in the MENA region, we collected and analyzed clinical, immunologic, and genetic data from countries in the region. The results of our analysis have important implications for prevention, prognosis, treatment, and allocation of resources.

## Methods

### Data Sources

The databases used included the Asian Pacific Society for Immunodeficiencies (APSID) registry, African Society for Immunodeficiencies (ASID) registry, Jeffrey Modell Foundation (JMF) registry, J Project centers, and International Consortium on Immune Deficiency (ICID) centers.

### MENA Inborn Errors of Immunity (MENA-IEI) Registry

Only patients with a clinical diagnosis of the IEI according to the criteria of the European Society for Immunodeficiencies (ESID) [1] were included in the MENA-IEI registry. Patient eligibility was based upon the highest pre-test probability of the highly qualified expert clinicians at participating centers. One of the following clinical indications was recommended: (i) suspected clinical diagnosis of IEI based on JMF 10 Warning Signs of inborn errors of immunity [9], (ii) suggested guidelines for non-infectious signs [13], (iii) newborn screen suggestive of IEI. A data form was distributed to the participating centers in corresponding countries. It included patients’ demographics, clinical diagnosis, and molecular diagnosis if available. It also included treatments used and survival status. Patients were stratified following the confirmed clinical and/or genetic diagnosis according to the International Union of Immunological Societies (IUIS) updated classification [3]. Physicians were given the opportunity to list “unspecified (new isolated immune phenotype)” or “other deficiencies (new associated immune syndrome)” for any additional disorders or gene mutations not listed in the IUIS classification.

According to the latest estimation (2020), MENA has a population of 578,000,000 citizens with a total DALY burden of diseases of 163,781,896 reported [10]. The geographical subregions have been assigned according to the Greater Middle East (GME) Variome Project with modifications [14], including the Turkish Peninsula (Turkey, plus Azerbaijan), Central/West Asia (Iran, Pakistan plus Afghanistan), Syrian Desert (Syria, Iraq, Kuwait, Jordan, Lebanon), Arabian Peninsula (Saudi Arabia, Yemen, Oman, United Arab Emirates/UAE, Qatar, Bahrain), and North Africa (Algeria, Tunisia, Morocco, Egypt, Libya plus Sudan). For various reasons, we were not able to obtain data from Armenia, Israel, Palestine, Western Sahara, Ceuta Melilla, and Mauritania. Clinical and immunological evaluations were coordinated by a clinician at a center participating in this study. For considering the rate of diagnosed patients with IEI in each country, we report the number of registered cases and calculated the point prevalence as the number of patients with IEI per 100,000 people in each country. On the data continuously collected from individual centers in participating countries since 2013, the trend in several aspects concerning complex care for patients with IEI was compared. This study received approval from the Ethics Committee of the Tehran University of Medical Sciences.

### Genetic Analysis and Diagnoses in IEI Patients

Targeted sequencing was performed on extracted genomic DNA from a selected group of patients with a confirmed clinical diagnosis or classical clinical presentations suggestive of a specific IEI and agreed to genetic testing [3]. For patients in whom targeted sequencing failed or had a clinical presentation resembling several genetic defects, whole-exome sequencing was performed to detect single-nucleotide variants, insertion/deletions and large deletions. Candidate variants were evaluated by the Combined Annotation Dependent Depletion (CADD) algorithm and an individual gene cutoff given by using the mutation significance cutoff (MSC) was considered for impact predictions [15]. The pathogenicity of all disease attributable gene variants was re-evaluated using the updated guideline for interpretation of molecular sequencing by the American College of Medical Genetics and Genomics criteria. Of note, no variants with uncertain significance (VUS) was considered as molecular definite diagnosis [16, 17].

After confirmation of their clinical and genetic diagnoses, patients were classified according to the IUIS updated classification. This classification includes nine categories of immunodeficiencies affecting cellular and humoral immunity (non-syndromic combined immunodeficiency or CID), combined immunodeficiencies with associated or syndromic features (syndromic CID and bone marrow failure), predominantly antibody deficiencies (PAD), diseases of immune dysregulation, congenital defects of phagocyte number or function (phagocytic disorders), autoinflammatory disorders, defects in intrinsic and innate immunity, complement deficiencies, and phenocopies of inborn errors of immunity [3]. According to the IUIS classification, immune dysregulation diseases comprise a group of disorders that can trigger defective or uncontrolled immune responses and are characterized by autoimmunity, due to dysregulation of adaptive immunity and/or hypersensitivity reactions due to defective lymphocyte tolerance. However, autoinflammatory disorders are defined as recurrent episodes of inflammation and autoimmune reactions due to hyperactivity of innate immune components and activation of pro-inflammatory cytokines [3, 4, 18]. Of note, DALY of immune-related disorder is a residual cause consisting of conditions that do not map to other causes within the blood disease hierarchy. This residual group consists mainly of rare immune disorders and blood disorders not resulting in anemia. From the International Statistical Classification of Diseases and Related Health Problems (ICD) chapter on immune disorders (the E chapter of Global Burden of Disease), 96 main disease codes were included (Table S1) [10]. To evaluate MENA countries’ impact on the identification of novel IEI genes and the causes of rare genetic diseases, patients from each country who were reported in the initial publication of these novel genes were compiled.

### Statistical Approach

Different parameters between patients’ groups and geographical regions were compared. One-sample Kolmogorov–Smirnov test was applied to estimate whether data distribution is normal. Parametric and non-parametric analyses were performed based on the finding of this evaluation. Statistical analysis was performed using SPSS (version 21.0.0, SPSS, Chicago, IL) and R statistical systems (version 3.4.1., R Foundation for Statistical Computing, Vienna, Austria). A *p*-value ≤ 0.05 was considered statistically significant.

## Results

A total of 17,120 patients with IEI (39.4% female) from 22 countries of the MENA region were included in this study: Turkey (*N* = 6392), Iran (*N* = 5373), Algeria (*N* = 1040), Tunisia (*N* = 710), Morocco (*N* = 693), Egypt (*N* = 667), Jordan (*N* = 544), Saudi Arabia (*N* = 502), Kuwait (*N* = 331), Oman (*N* = 185), Azerbaijan (*N* = 139), Qatar (*N* = 137), Libya (*N* = 106), Pakistan (*N* = 103), Sudan (*N* = 72), Lebanon (*N* = 57), UAE (*N* = 30), Syria (*N* = 15), Afghanistan (*N* = 9), Iraq (*N* = 8), Yemen (*N* = 4), and Bahrain (*N* = 3, Table 1). The median male/female gender ratio was 1.3 (range 0.7 in Syria–2.3 in Jordan). Parental consanguinity was documented in 60.5% of cases (range 34.7% in Turkey–79.7% in Egypt, Table 2). Moreover, 27.3% of patients were from multi-case families with a confirmed previous family history of IEI. The median (interquartile range/IQR) age of the patients at the onset of disease was 36 (12–66) months. As expected, only 546 cases (3.2%) showed the first disease manifestation after 15 years of age.

**Table 1:** Demographic data about 22 participated countries from the Middle East and North Africa (MENA) region regarding the rate of inborn errors of immunity (IEI) diagnosis and total disability-adjusted life year (DALY) and mortality rates of children under age 15 years [10]

Region/country	Population	Population growth rate	Births/1000 population	DALY rate/100 K < 15 years	Death rate/100 K < 1 year	Death rate/100 K 1–4 years	Death rate/100 K 5–14 years	Registered IEI patients	IEI prevalence/100 K
Afghanistan	39,348,000	2.30%	32.49	50,776.31	4803.73	257.18	81.6	9	0.02
Algeria	43,851,000	1.63%	21.5	18,667.41	1755.63	62.01	33.81	1034	2.36
Azerbaijan	9,981,000	0.91%	15.8	22,377.57	2632.66	88.24	40.46	138	1.38
Bahrain	1,729,000	2.19%	13.1	8345.43	521.65	32.54	20.27	3	0.17
Egypt	98,420,000	2.38%	28.8	15,009.32	1241.74	75.33	38.3	667	0.68
Iran	83,992,000	1.19%	17.4	12,231.01	957.28	40.13	32.9	5384	6.41
Iraq	40,222,000	2.50%	30	16,096.74	1272.4	82.37	40.48	8	0.02
Jordan	9,956,000	2.02%	23.6	14,051.47	1324.71	49.38	27.87	544	5.46
Kuwait	4,270,571	1.38%	18.8	10,530.62	780.15	36.14	20.25	331	7.75
Lebanon	6,825,000	-3.13%	14.4	10,983.94	805.52	34.95	18.83	57	0.83
Libya	6,408,000	1.45%	17.2	12,982.67	824.23	128.36	35.62	106	1.65
Morocco	36,910,000	0.95%	17.5	16,280.34	1611.43	61.79	29.59	689	1.87
Oman	4,829,000	2.00%	23.7	12,635.83	835.39	52.05	30.63	185	3.83
Pakistan	2.21E + 08	2.00%	28.25	52,689.67	5561.54	281.18	97.03	103	0.05
Qatar	2,782,000	1.95%	9.5	9286.73	660.1	31.56	21.95	137	4.92
Saudi Arabia	31,560,000	1.63%	15.6	7830.88	450.47	31.89	15.85	502	1.59
Sudan	2,166,000	2.93%	34.2	36,033.86	3517.71	242.79	53.06	72	3.32
Syria	17,500,000	n/a	20.7	14,728.66	960.83	111.88	45.42	15	0.09
Tunisia	11,270,000	0.95%	17.4	11,451.91	945.26	49.11	25.02	710	6.3
Turkey	84,339,000	0.49%	15.4	13,796.04	1252.02	75.7	22.71	6392	7.58
UAE	9,397,600	1.44%	9.8	7350.14	376.65	28.3	19.32	30	0.32
Yemen	29,825,000	2.17%	27.6	40,778.49	3995.52	232.01	82.19	4	0.01
MENA region	578,000,000	1.56%	20.98	21,730.78	2060.17	114.11	42.29	17,120	2.96
Global [2, 9]	7,800,000,000	1.1%	18.5	30,031.53	2844.75	242.80	52.20	180,000	2.31

*UAE* United Arab Emirates, *100 K* 100,000 population

**Table 2:** Gender, consanguinity, and number of patients with different inborn errors of immunity entities (based on International Union of Immunological Societies, IUIS classification) among the Middle East and North Africa region countries

Region/country	Male/female ratio	Consanguinity (%)	IUIS classification								
			PAD	CID	Syndromic CID	Phagocytic defects	Complement deficiencies	Immune dysregulation	Autoinflammations	Innate immune defects	Phenocopies
Afghanistan	1.3	66.7	5	3	NR	1	NR	NR	NR	NR	NR
Algeria	1.5	49.2	301	363	197	88	22	45	7	11	6
Azerbaijan	2.0	56.4	63	9	37	5	NR	23	1	NR	1
Bahrain	2.0	66.6	2	1	NR	NR	NR	NR	NR	NR	NR
Egypt	1.4	79.7	124	223	99	91	7	42	69	12	NR
Iran	1.5	60.1	1488	684	926	894	114	119	1013	134	1
Iraq	1.0	62.5	3	4	NR	NR	NR	NR	NR	NR	1
Jordan	2.3	60.0	38	70	19	55	NR	39	323	NR	NR
Kuwait	1.1	78.0	59	99	79	21	23	47	1	2	NR
Lebanon	0.9	48.8	24	11	9	6	NR	4	1	2	NR
Libya	1.6	62.5	13	31	19	18	NR	9	15	1	NR
Morocco	1.3	43.2	171	133	156	112	23	28	32	34	4
Oman	1.6	76.0	33	38	35	59	7	8	NR	5	NR
Pakistan	1.8	75.9	51	23	11	17	1	NR	NR	NR	NR
Qatar	1.3	61.1	40	24	34	16	3	13	NR	7	NR
Saudi Arabia	1.1	75.0	62	300	31	47	29	30	NR	3	NR
Sudan	2.0	66.6	29	43	NR	NR	NR	NR	NR	NR	NR
Syria	0.7	60.0	7	2	1	1	1	2	1	NR	NR
Tunisia	1.4	58.2	126	203	161	180	3	34	NR	3	NR
Turkey	1.6	34.7	4423	375	471	279	51	72	650	71	NR
UAE	1.2	67.0	5	11	4	6	3	1	NR	NR	NR
Yemen	1.0	50.0	3	1	NR	NR	NR	NR	NR	NR	NR
MENA region	1.3	60.5	7070	2651	2289	1896	287	516	2113	285	13

*PAD* predominantly antibody deficiencies, *CID* combined immunodeficiencies, *UAE* United Arab Emirates, *NR* not reported

The median delay in diagnosis was 41 months (range 2 days–51 years) and remained almost unchanged since identifying the first patient of the cohort in 1984 (*r* =  − 0.002, *p* = 0.25). The majority of patients with a delay in diagnosis longer than 10 years have been identified after 2000 (Figure S3) and mainly included antibody deficiency (43.3%) and immune dysregulation (20.6%). The delayed diagnosis was slightly less frequent in patients with parental consanguinity compared to those with no consanguinity (39 [IQR 30–45] months vs. 43 [IQR 32–52] months, *p* = 0.04), but was similar between sporadic and multi-case families (37 [IQR 29–48] months vs. 36 [IQR 28–49] months, *p* = 0.52). There was a shorter delay in diagnosis in cases with a known genetic diagnosis compared to unsolved cases (31 [IQR 27–35] months vs. 48 [IQR 39–58] months, *p* = 0.002).

**Fig. 1:**
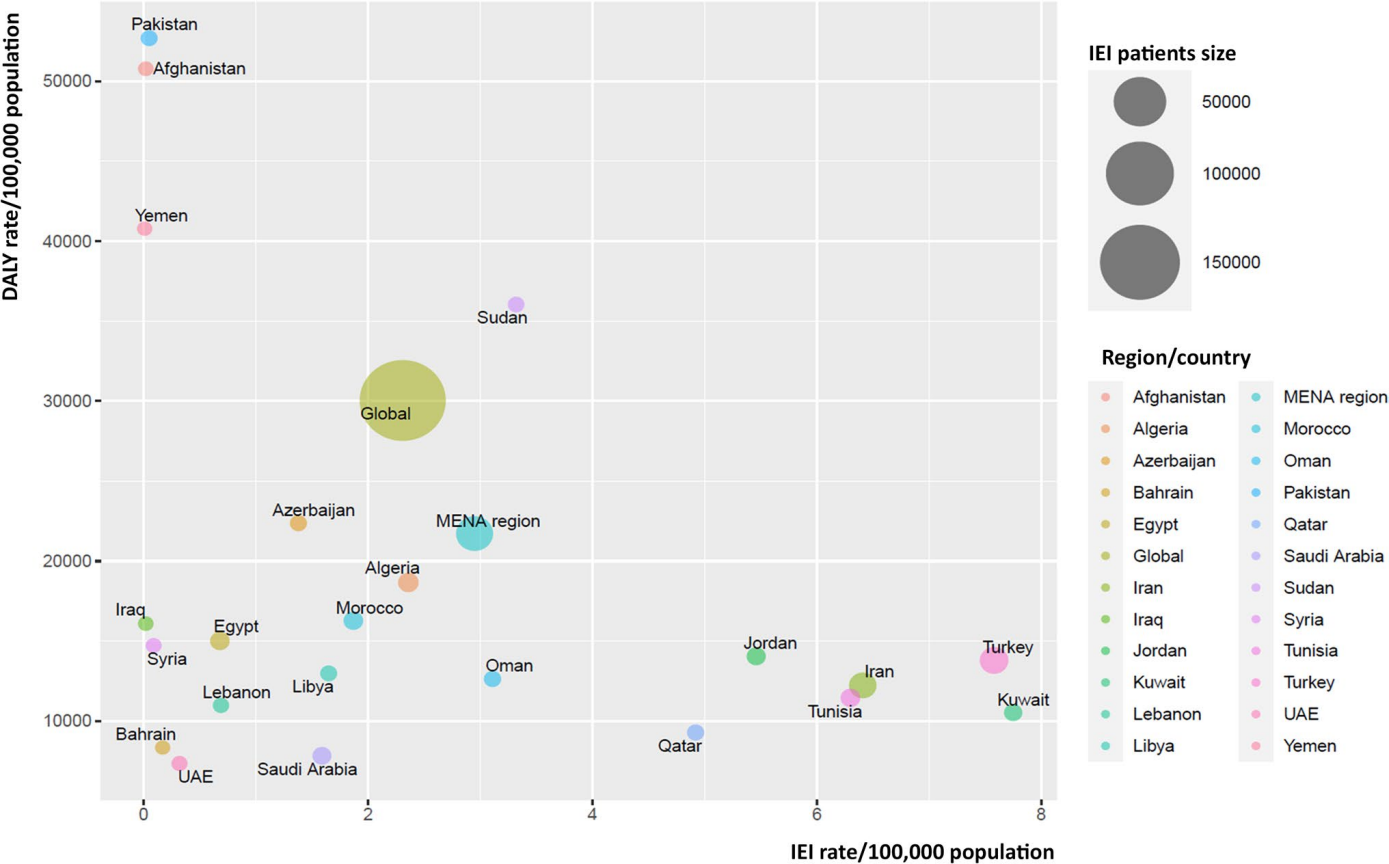
The association of rate of inborn errors of immunity (IEI) diagnosis and rate of disability-adjusted life year (DALY) [10] in children within countries from the Middle East and North Africa region

Improvement in DALY and death rate for children were reported in all studied countries during 1990–2019 (Figure S2). Of note, the highest rate of DALYs and death rate for children were recorded in countries with the lowest rate of registered IEI patients, namely Pakistan, Afghanistan, and Yemen (*r* =  − 0.35, *p* = 0.09, Table 1, Fig. 1). Disease-specific DALY analysis of children in MENA countries showed that this reverse association is more intense for lower respiratory tract infections (*r* =  − 0.37, *p* = 0.07), chronic enteropathies (*r* =  − 0.37, *p* = 0.07), and neoplasms (*r* =  − 0.27, *p* = 0.19, Figure S4). DALYs of atopic/allergic disease and upper respiratory infection do not have any association with the level of IEI diagnosis in these countries. Countries with higher DALY of immune-related disease also have a higher detection rate of IEI (*r* = 0.25, *p* = 0.24), suggesting varied immunologic knowledge and facility capacities in the region (Figure S4).

**Fig. 2:**
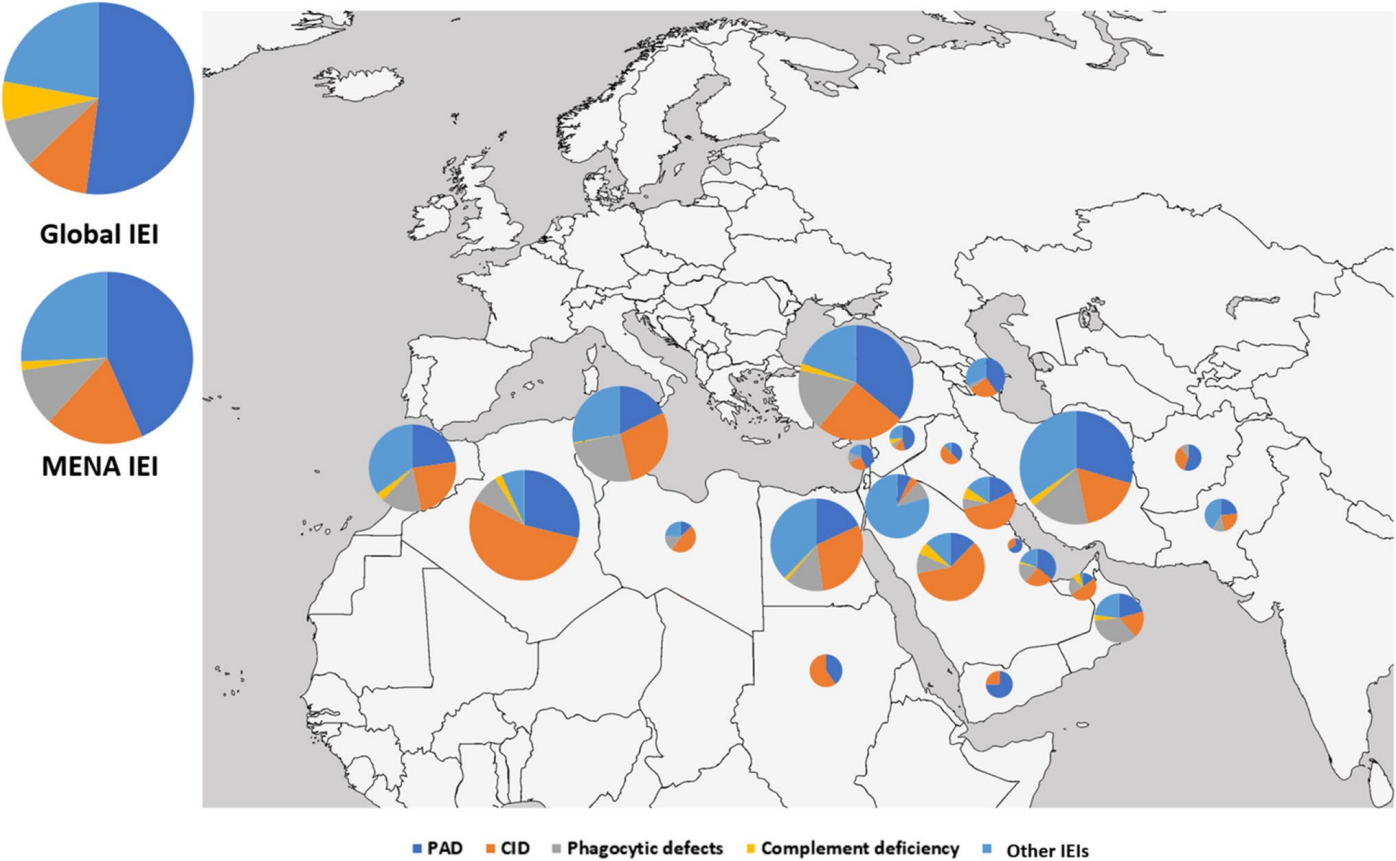
Distribution of different inborn errors of immunity (IEI) entities among different countries in the Middle East and North Africa (MENA) region. PAD, primary antibody deficiency; CID, combined immunodeficiency. Global data has been retrieved from references (for more details, please see Table 2) [2, 9]

According to different IEI categories, patients’ distribution was also significantly variable between participating countries, reflecting variable awareness of specific diseases, different genetic backgrounds/ethnicities, and uneven diagnostic resources. Combined immunodeficiencies were the dominant disorders in North African countries and the Arabian Peninsula (mainly in Saudi Arabia and Sudan with a frequency of 60%), while primary antibody deficiencies were more frequent in Central/West Asia, the Turkish Peninsula, and the Syrian Desert (mainly in Turkey with a frequency of 70%) (Fig. 2, Table 2). There was no registered patient with unspecified or other deficiencies in our cohort. The most common clinical diagnoses recorded in the MENA region were familial Mediterranean fever (FMF, *n* = 2042, 11.9%), common variable immune deficiency (CVID, *n* = 1935, 11.3%), severe combined immunodeficiency (SCID, *n* = 1524, 8.9%), non-syndromic combined immunodeficiency (CID, *n* = 1028, 6.0%), ataxia-telangiectasia (A-T, *n* = 891, 5.2%), chronic granulomatous disease (CGD, *n* = 823, 4.8%), hyper IgE syndrome (HIES, *n* = 789, 4.6%), agammaglobulinemia (*n* = 755, 4.4%), symptomatic selective IgA deficiency (SIgAD, *n* = 719, 4.2%), and major histocompatibility complex class II deficiency (MHC-II, *n* = 702, 4.1%).

**Fig. 3:**
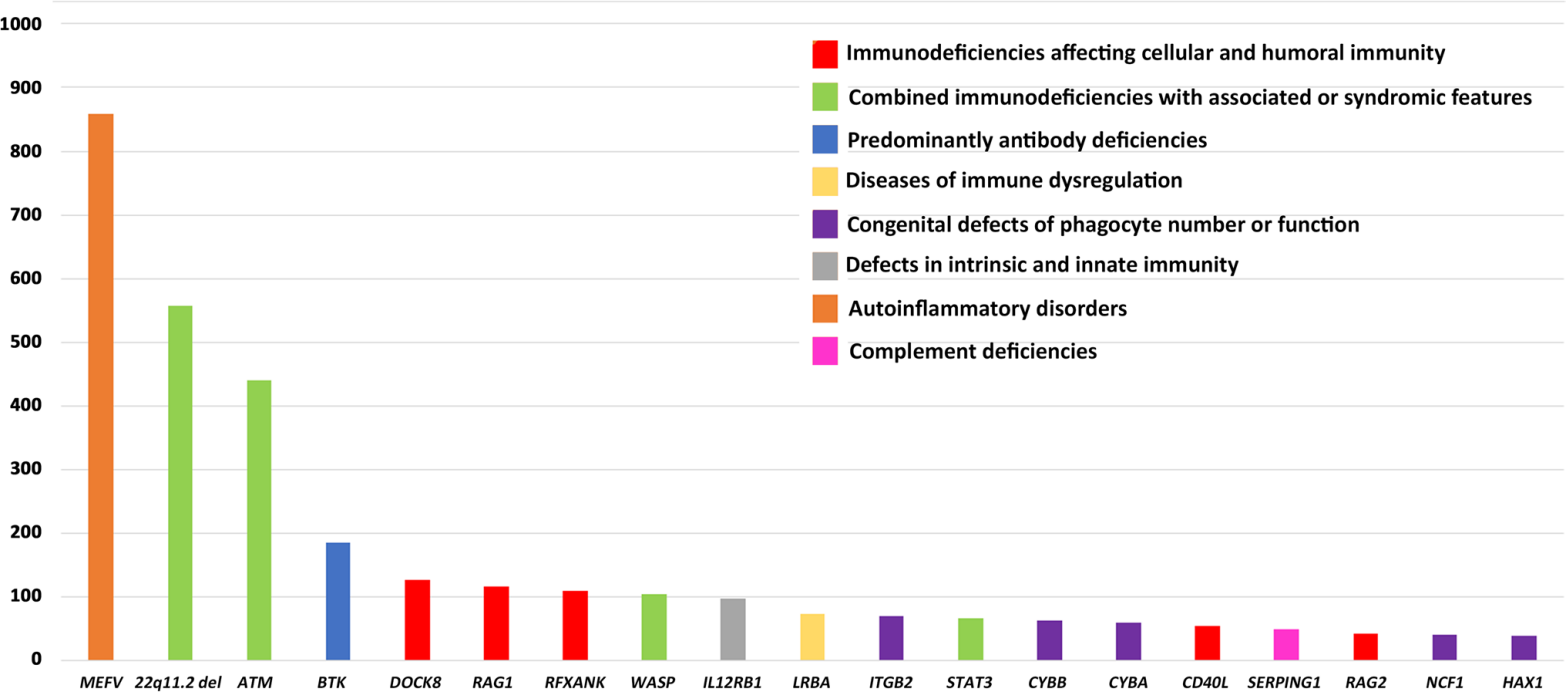
Number of patients with frequent genetic defects in the Middle East and North Africa cohort classified based on International Union of Immunological Societies (IUIS) categories of inborn errors of immunity (only genetic defects with > 40 patients are shown)

Among all reported patients included in this study, 5871 patients (34.3%) were genetically evaluated. The diagnostic yield was 83% (72.6% with pathogenic variants and 10.4% with likely pathogenic variants). As expected, most patients with genetic diagnoses had autosomal recessive inheritance (*n* = 3181, 65.2%), whereas X-linked recessive and autosomal dominant inheritances were less frequent (16.7% and 17.9%, respectively). The most frequent genetic defects identified were *MEFV* variants (*n* = 859, 13.1%), 22q11.2 deletion (*n* = 558, 8.5%), and *ATM* variants (*n* = 440, 6.7%), respectively (Fig. 3). Of note, 142 genes of the IEI genes known to date [3, 4] (31.4%, Table S2) have been initially identified in patients originating from the MENA region indicating the impact of genetic evaluation in this specific geographical region of the world. Detailed analysis of 1339 unsolved cases revealed that patients with a clinical diagnosis of CVID (48.6%), non-syndromic CID (27.8%), and HIES (18.0%) have more probability to remain with unknown genetic defects and are probably candidates for the identification of novel genetic defects.

We next evaluated the trend of clinical and molecular diagnosis in the region across time by comparing our cohort with the MENA cohorts in the 2013 and 2018 JMF reports [8, 19]. The number of reporting countries doubled (from 11 countries in 2013, Kuwait, Qatar, Oman, Turkey, Egypt, Tunisia, Saudi Arabia, Morocco, Azerbaijan, and Jordan) to 22 countries in 2020 with the addition of Algeria, Libya, Pakistan, Sudan, Lebanon, UAE, Syria, Afghanistan, Iraq, Yemen, and Bahrain. The number of diagnosed cases increased from 9133 in 2013 to 10,916 in 2018 and 17,120 in 2020, indicating an increase of 87% during the last 7 years and of 56.8% during the last 2 years. Moreover, genetic diagnosis increased from 604 in 2013 to 1039 in 2018 and to 4873 in 2020. Improved genetic diagnosis was more striking for syndromic CIDs and autoinflammatory disorders (Figure S5).

Only 47 patients have been identified by newborn screening in this cohort (< 0.3%, Iran, Turkey, Algeria, Saudi Arabia, Qatar, and UAE). Non-infectious clinical manifestation was the initial presentation in 3002 cases (17.5%), mainly in cases with immune dysregulation (*n* = 502, 97.2%), complement deficiency (*n* = 263, 91.6%), and syndromic CID (*n* = 1166, 50.9%, Table 3). Autoimmunity was documented in 18.3% of all patients (in 68.4% of cases with autoinflammatory disorders and 61.5% of cases with phenocopies of IEIs). Atopic disorders were present in 14.3% of all patients (in 73.1% of patients with immune dysregulation), lymphoproliferative disorders in 13.1% (in 84.4% of patients with immune dysregulation), enteropathy in 7.6% (in 53.4% of patients with immune dysregulation), and malignancies in 2.3% (in 38.4% of patients with IEI phenocopies).

**Table 3:** Presenting manifestations and non-infectious complications in different inborn errors of immunity entities (based on International Union of Immunological Societies, IUIS classification) among the Middle East and North Africa region countries

Clinical manifestations	PAD (%)	CID (%)	Syndromic CID (%)	Phagocytic defects (%)	Complement deficiencies (%)	Immune dysregulation (%)	Autoinflammations (%)	Innate immune defects (%)	Phenocopies (%)	Total IEI (%)
Presenting features										
LRI	3207 (45.3)	1219 (45.9)	668 (29.1)	519 (27.3)	34 (11.8)	123 (23.8)	602 (28.4)	85 (29.8)	8 (61.5)	6465 (37.8)
GI	219 (3.1)	814 (30.7)	310 (13.5)	217 (11.4)	28 (9.7)	184 (35.6)	511 (24.1)	260 (91.2)	1 (7.6)	2544 (14.8)
Vaccine side effects	19 (0.2)	95 (3.6)	14 (0.6)	38 (2.0)	NR	2 (0.3)	NR	49 (17.2)	NR	217 (1.2)
Other severe infections*	624 (8.8)	628 (23.6)	337 (14.7)	517 (27.3)	88 (30.7)	49 (9.5)	9 (0.4)	78 (27.3)	5 (38.4)	2335 (13.6)
Non-infectious	349 (4.9)	440 (16.6)	1166 (50.9)	209 (11.0)	263 (91.6)	502 (97.2)	27 (1.2)	41 (14.3)	5 (38.4)	3002 (17.5)
Non-infectious complications during the follow-up										
Autoimmunity**	674 (9.5)	346 (13.0)	228 (10.0)	69 (3.6)	55 (19.1)	272 (52.7)	1446 (68.4)	32 (11.2)	8 (61.5)	3130 (18.3)
Allergy	753 (10.6)	254 (9.6)	921 (40.2)	64 (3.4)	27 (9.4)	377 (73.1)	29 (1.4)	25 (8.8)	1 (7.7)	2451 (14.3)
Enteropathy	413 (5.8)	208 (7.8)	148 (6.5)	5 (0.2)	NR	276 (53.4)	196 (9.3)	57 (17.5)	NR	1303 (7.6)
Lymphoproliferative	612 (8.6)	327 (12.3)	354 (15.5)	318 (16.8)	9 (3.1)	436 (84.4)	136 (6.4)	50 (6.4)	2 (15.4)	2244 (13.1)
Malignancy	88 (1.2)	56 (2.1)	153 (6.7)	38 (2.0)	NR	51 (9.8)	16 (0.7)	NR	5 (38.4)	407 (2.3)

*PAD* predominantly antibody deficiencies, *CID* combined immunodeficiencies, *NR* not reported, *LRI* lower respiratory infections as the first presentation, *GI* gastrointestinal infections and chronic diarrhea as the first presentation, *Vaccine side effects* BCG or oral polio vaccine side effects as the first presentation

^*^Other severe infections as the first presentation of patients include sepsis, septic arthritis, osteomyelitis, stomatitis, urine tract infection, nephritis, cellulitis, blepharitis, folliculitis, skin ulcer and abscesses, periodontitis, omphalitis, meningitis, and encephalitis

^**^Autoimmunity include autoimmune hemolytic anemia, immune thrombocytopenia, autoimmune neutropenia, pancytopenia, Evans syndrome, pernicious anemia, autoimmune enteropathies, ulcerative colitis, Crohn’s disease, celiac disease, autoimmune vasculitis, autoimmune glomerulonephritis, autoimmune thyroiditis, autoimmune polyarthritis, rheumatoid arthritis, psoriasis, systemic lupus erythematosus, Addison’s disease, scleroderma, Sjögren’s syndrome, myasthenia gravis, autoimmune serositis, autoimmune myositis, autoimmune uveitis, panniculitis, vitiligo, alopecia areata, type 1 diabetes mellitus, Guillain–Barre syndrome, autoimmune polyendocrine syndrome

Immunoglobulin (Ig) replacement and hematopoietic stem cell transplantation (HSCT) therapies were compared in 2020 with those of 2013 and 2018. Compared to 2013, there has been a 7.3-fold increase in the number of patients receiving Ig replacement and a 2.0-fold increase in the number of patients undergoing HSCT (Figure S6). Ig replacement therapy was administered mainly for PAD (46.1%), non-syndromic CID (28.0%), and syndromic CID (18.6%) patients. HSCT was prioritized for cases suffering from non-syndromic CID including SCID (40.4%), syndromic CID (19.1%), and immune dysregulation (15.6%). Of note, less than 1% of patients are receiving subcutaneous Ig replacement in the MENA region.

The median follow-up period for registered patients was 67 (IQR 45–73) months at the time of the study, with 3.7% of patients assigned as censored during the follow-up period. Of note, 15.8% of cases (*n* = 2704) died due to IEI complications at a median age of 62 [IQR 12–78] months. Males accounted for 61.5% of the deaths. Causes of mortality were mainly sepsis (53.1%) and respiratory failure (27.6%). Fifty-two patients died due to complications of HSCT. They represented 12.7% of the total transplanted patients with follow-up. The mortality rate was the highest in the patients with non-syndromic CID (51.7%, median age: 3.5 years) and syndromic CID (22.5%, median age: 6 years) and was lowest among patients with autoinflammatory (0.8%, median age: 54 years) and PAD (5.8%, median age: 12.5 years). Of note, the mortality rate was significantly higher in multi-case families compared to sporadic patients (21.1% vs. 11.3%, *p* < 0.001). We further evaluated the death rate and median age at death within the group of patients with a molecular diagnosis. Specific genes associated with CID and phagocytic defects were among the most life-threatening mutations. There was 63.3% mortality in RFXANK deficiency (median 16 months), 24.7% in RAG1 deficiency (median 22 months), 25.6% in IL2RG deficiency (median 12.5 months), 21.4% in ITGB2 deficiency (median 11 months), and 10.9% in CYBB deficiency (median 183 months).

## Discussion

With the inclusion of 17,120 patients with IEI from 22 countries, this is the most extensive registry from the MENA region with diverse ethnic origins. There was a strikingly high yield of 83% in achieving a molecular diagnosis. This carries vital implications for the targeted treatment and future management of IEI diseases in these countries.

We have recently reviewed systematically the IEI diagnosis globally (Table S3) [20]. The MENA-IEI registry of 17,120 patients is the second-largest regional registry after the ESID registry (*n* = 33,710), which overlaps with our registry in that it contains data from Iran, Egypt, and Turkey [21]. Other large regional registries are the Latin American Society for Immunodeficiencies (LASID, *n* = 8176) [22] and the United States Immunodeficiency Network (USIDNET, *n* = 5485)[23]. Of note, the estimated frequency of IEI might be even higher than that reported in the current registry due to the unavailability of data from all centers in the country and lack of a national registry. From 2013 to 2020, we observed an 87% increase in patients’ numbers from the MENA region. This is still below the reported global increase of 133% during the same period [8, 9]. Data from other regions are available from 2013 through 2018. Our study shows an increase of 19.6% in the MENA region compared to 1961% in Australia, 64.0% in Latin America, and 32.7% in the USA [19]. Only 3.2% of IEI patients in the MENA countries present in adulthood; however, this rate is around 23.8% in ESID, 32.0% in USIDNET, and 36.1% in the global report of JMF [9, 19–21, 23]. The mortality rate of IEI patients in the MENA region was 15.8% which is higher than the rate reported by USIDNET (8.7%) [23] and ESID (9.2%) [21]. We observed a 1.3 higher male/female ratio; although this ratio is equivalent to global (1.5), ESID (1.25), and USIDNET (1.45) ratios, it is lower than the LASID registry ratio (2.5) [20–23].

A salient feature of this study is the high yield of molecular diagnosis, which was achieved in 83% of the patients examined. This diagnostic yield is higher than the reported global rate of 13.2% [20]. Moreover, this rate is higher than the recently reported rate of 56% from the MENA region [14, 24, 25] and 40% from Western countries [26, 27] that like our study included all IEI entities. The high diagnostic yield in the MENA region is likely due to the high incidence of parental consanguinity in the IEI population of patients studied (60.5%), itself a reflection of the high prevalence of consanguineous marriages (20–50%) in the MENA region. The high rate of molecular diagnosis has a practical implication for the treating physicians toward appropriate clinical management, prenatal diagnosis, and targeted therapy. Prenatal diagnosis in childhood-onset patients has an important significance apart from decreasing the burden of diseases, because the parents and also the first-degree relatives of patients are still at childbearing age and would benefit from genetic counseling and preimplantation diagnosis [25, 28]. Of note, 10.4% of these genetic defects were likely-pathogenic and expression assays of the encoding protein or in vitro functional laboratory data of the upstream and downstream pathways are essential mainly for novel missense mutations. Subsequently, the comparison of diagnostic yield in this study and other published global cohorts needs to be revised after this evaluation in the future. Correlation of the identified genetic pathogenic variant with the clinical and immunological phenotype of individual affected patients is important for making prognostic and treatment decisions.

One of the main improvements in the MENA region is the increase in the fraction of patients receiving intravenous Ig replacement therapy. However, there has been a lack of resources for the performance of newborn screening and availability of genetic tests for all patients. Moreover, other shortages in the region are the unavailability of subcutaneous Ig treatment which is important for patients who have poor venous access or do not have access to a hospital, and unattainability to HSCT for patients who need this therapeutic modality. These limitations may underlie the relatively higher mortality rate of patients with IEI in the MENA region compared to USIDNET [23] and ESID [21]. More efforts should be made to introduce personalized therapies for patients with IEI in this region [28]. This can be done by better characterizing the molecular defect and targeted therapies to improve disease outcomes. Clinical immunologists in the region are still struggling with the standardization and expansion of newborn screening programs for CIDs and antibody deficiency. In addition, pre-symptomatic diagnosis of patients with other IEIs that carry a high rate of morbidity and mortality, including phagocytic and complement deficiency, should also move forward in MENA countries [29]. Increasing awareness about IEI and improved diagnostic capabilities of IEI will result in increased referrals for HSCT. This necessitates the allocation of resources to expand the currently limited capacity for transplantation and the training of young physicians to provide such service to IEI patients.

Discrepancies are observed in the diagnostic rate in some countries with the highest childhood DALY and mortality rates. These can be improved by sharing consensus clinical guidelines and experiences between neighboring countries as well as exchange visits between countries in the MENA region. Other collaboration possibilities include help with genetic diagnosis, IEI prenatal diagnosis, and treatment for IEI patients from countries with limited resources. The economic burden of IEIs through both the direct and indirect costs represents a major challenge on the patients, their families, and societies since they are relatively prevalent in the MENA region compared to other regions. Hence, continuing the study of the IEI epidemiology should be emphasized to provide feedback to healthcare authorities and policymakers to improve diagnosis and management of these patients.

This MENA-IEI registry aims to continue the collaboration between clinicians and basic scientists from the region and to enrich the existing collaborations with international research centers. Collection of a comprehensive pool of genetic data on patients from the MENA region will allow better prognostic and therapeutic studies to be conducted in the future. Furthermore, it will help define the spectrum of specific rare genetic immune disorders, will promote the discovery of novel gene defects that underlie IEIs, and will provide a better understanding of the pathophysiology of known IEIs. This has important implications for individual patients and for the community. On the one hand, it improves delivery of personalized care. On the other hand, it helps understand the mechanisms of common diseases such as cancer, allergy, and autoimmunity that often arise in patients with IEI.

## Conclusion

This report represents the first step in providing valuable information on the MENA-IEI registry. Further information is needed to gain a better understanding of IEI in this region in order to guide efforts at prevention and treatment and provide a sound rationale for the allocation of public health resources.

## Supplementary Information

Below is the link to the electronic supplementary material.

Supplementary file1 (PDF 1140 KB)

## Data Availability

The raw data supporting the conclusions of this article will be made available by the authors, without undue reservation, to any qualified researcher.
