# Supplementary material for: Consensus Middle East and North Africa Registry on Inborn Errors of Immunity
Source: J Clin Immunol. 2021 May 29;41(6):1339–51. doi: 10.1007/s10875-021-01053-z (PMC8310844; doi:10.1007/s10875-021-01053-z)
Supplement: Supplementary file 1 — Supplementary file1 (PDF 1140 KB) [file 10875_2021_1053_MOESM1_ESM.pdf]

**Supplementary Data**

## Consensus Middle East and North Africa Registry on Inborn Errors of Immunity

**Figure S1-** Compare Map of global all-cause disability-adjusted life years (DALYs) rate for 2019 and 1990 for both sexes below age 15 years [10].

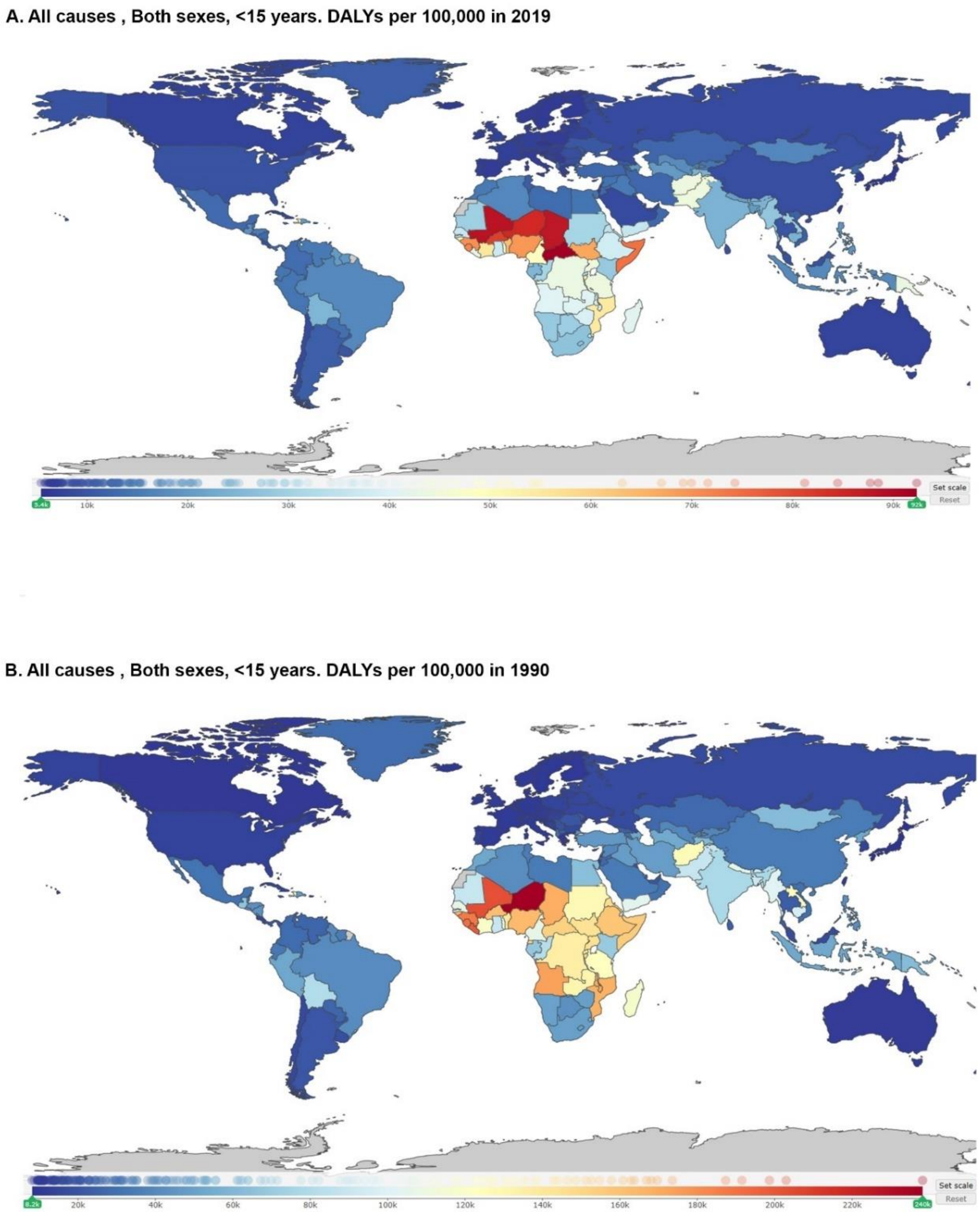

**Figure S2-** The trends of all-cause mortality rate and disability-adjusted life years (DALYs) rate from 2019 until 1990 for both sexes [10]. (A, B) Comparing Middle East and North Africa region with overall Global measures in three age categories of <1year, 1-4 years and 5-14 years. (C, D) Comparing different countries in the MENA regions.

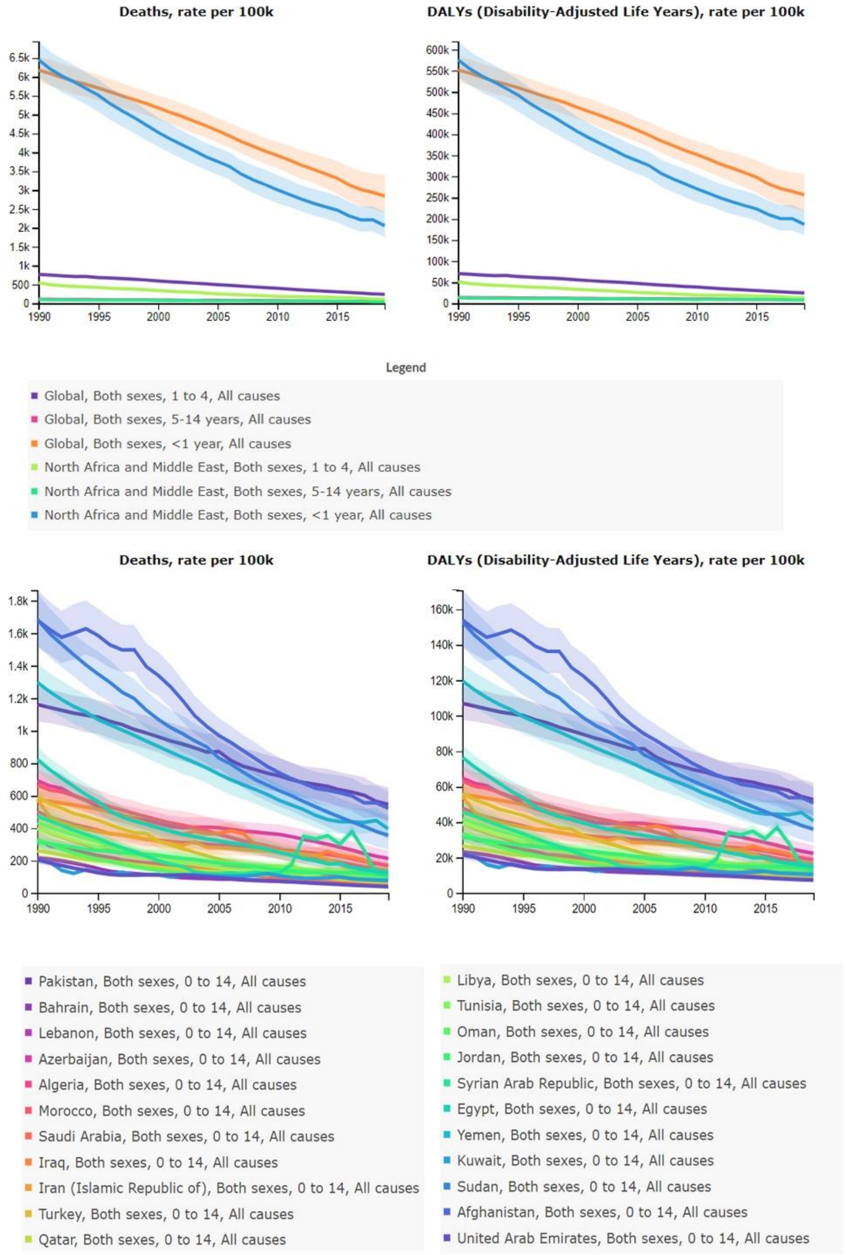

**Figure S3-** Trend of diagnosis of Inborn Errors of Immunity patients in the Middle East and North Africa region during 1984-2020. Delay in diagnosis was counted as lag between the onset of the first sign/symptom of the disease and the time of complete Inborn Errors of Immunity clinical diagnosis. Each dot represents one patient, blue line depicts the linear regression model and the gray zone accounted for 95% confidence interval.

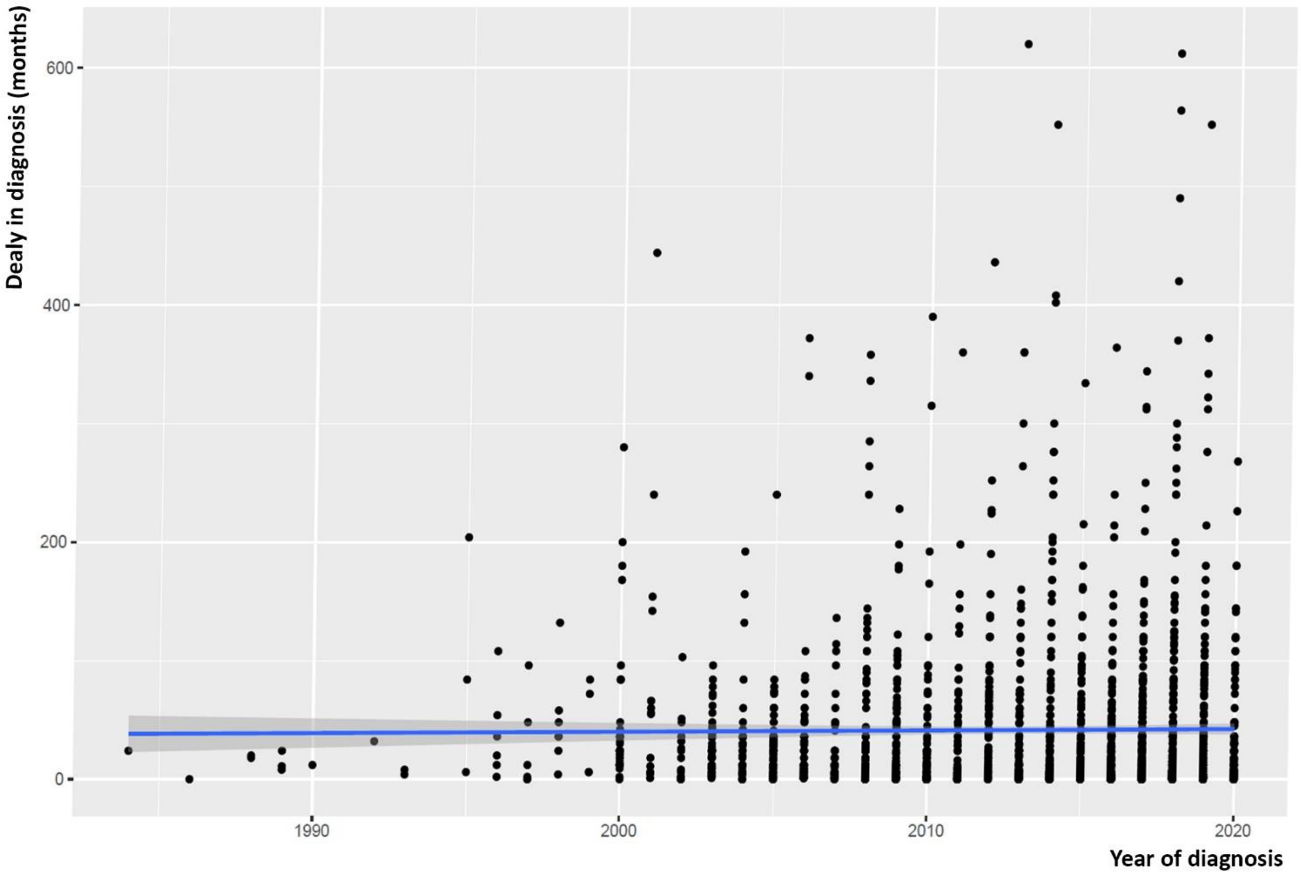

**Figure S4-** The association of rate of Inborn Errors of Immunity diagnosis and disease specific disability-adjusted life year (DALY)[10] of children including (A) upper respiratory infections, (B) lower respiratory infections, (C) enteropathies, (E) atopic disorders, (F) neoplasms and (D) other immune-related disorders in countries from Middle East and North Africa region.

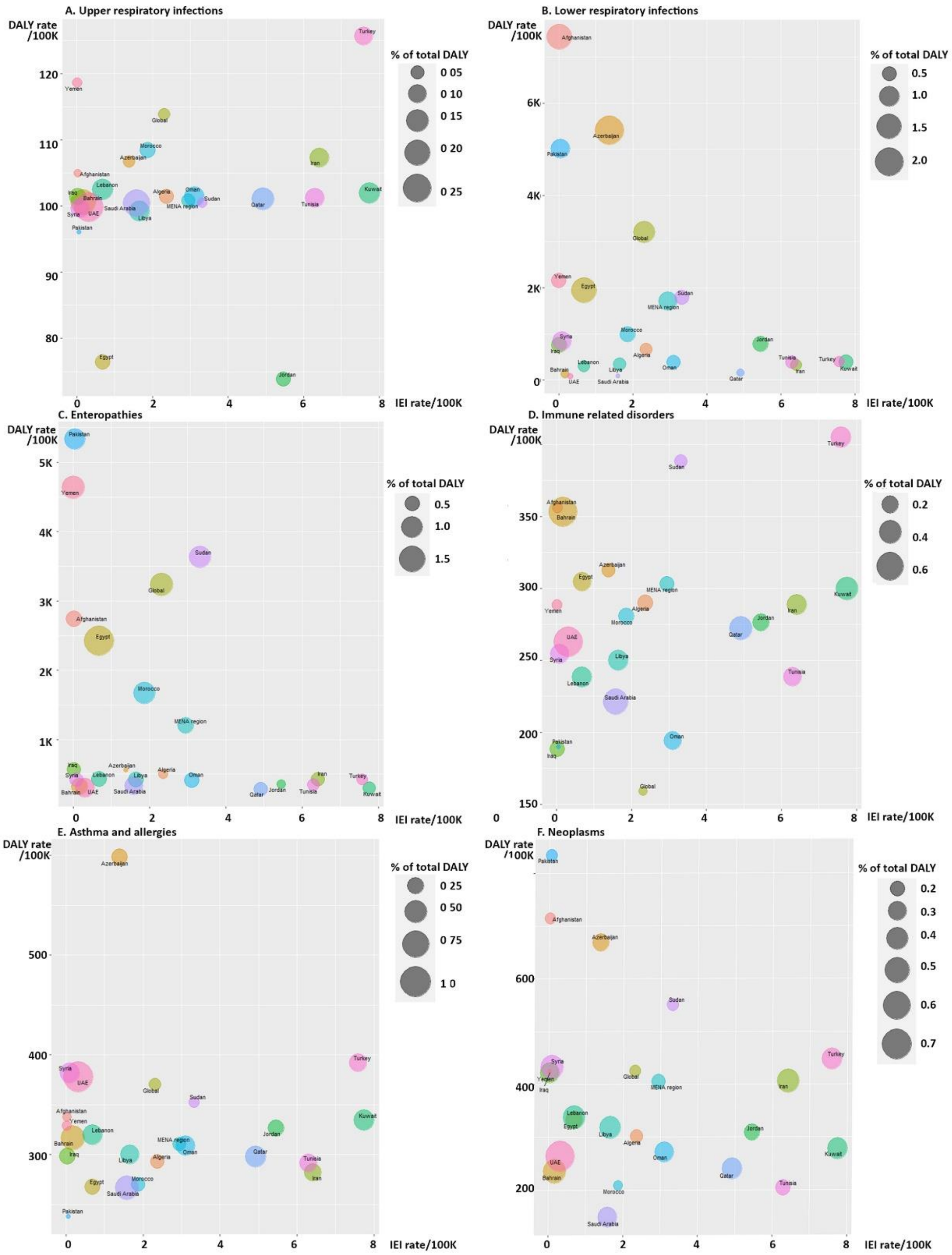

**Figure S5-** Trends of (A) clinical and (B) molecular diagnoses of Inborn Errors of Immunity in the Middle East and North Africa region during 2013-2020.

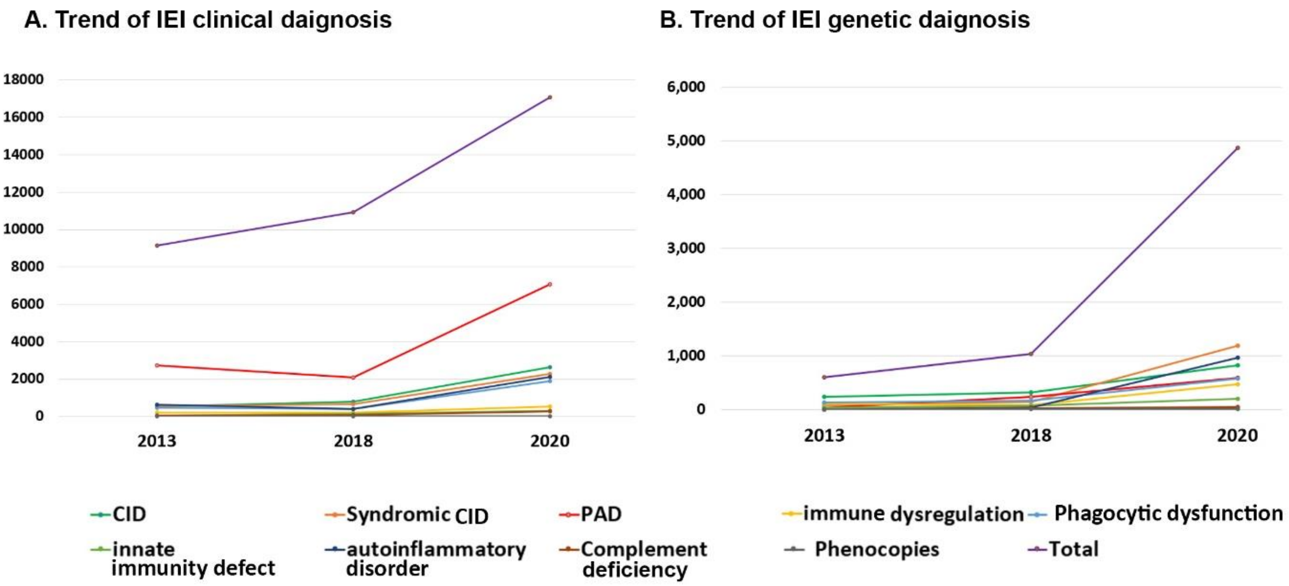

**Figure S6-** Trends of (A) immunoglobulin replacement and (B) hematopoietic stem cell transplantation therapies in the Middle East and North Africa region for Inborn Errors of Immunity patients during 2013 (11 countries registered) - 2020 (22 countries registered).

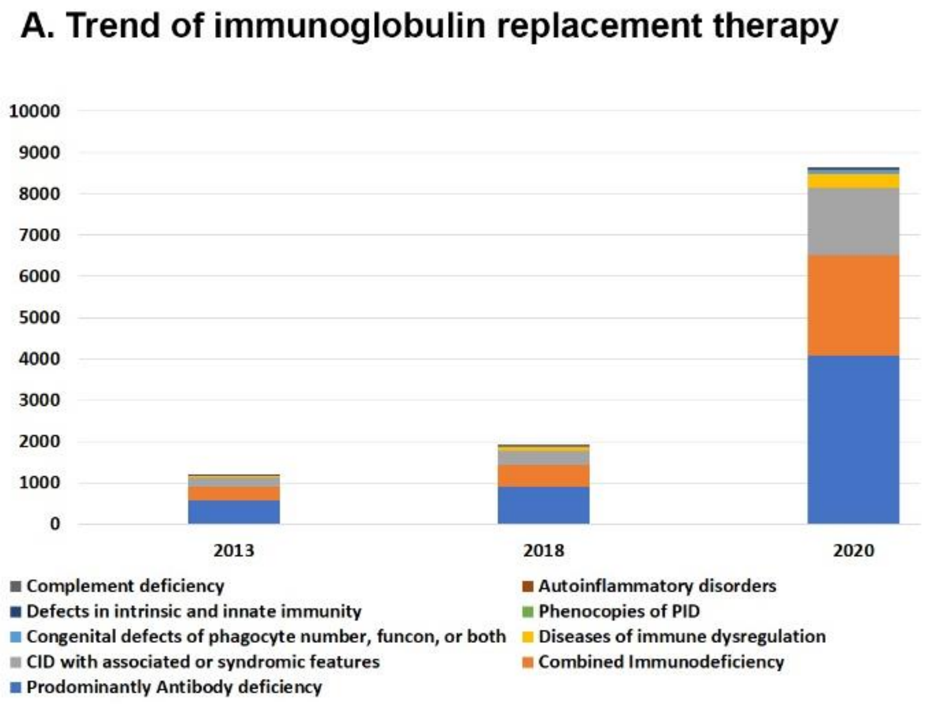


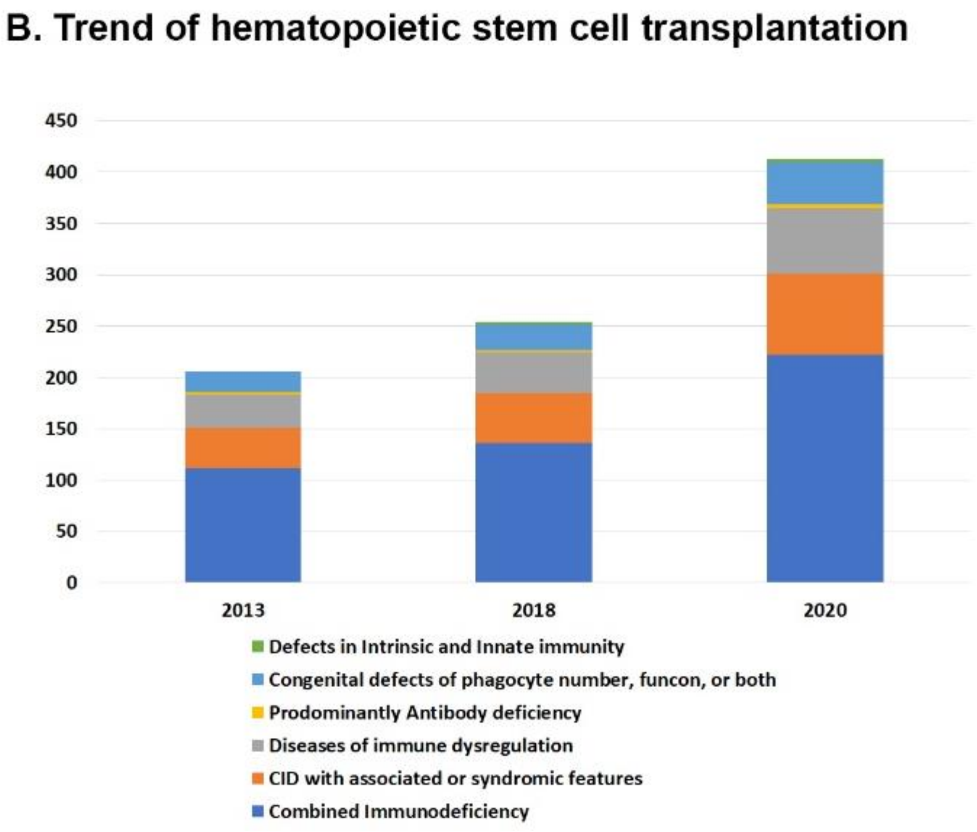

**Table S1-** International Statistical Classification of Diseases and Related Health Problems (ICD) code of immune-related disease included in the E chapter of Global Burden of Disease [10].

| **D codes group** |
| --- |
| D64.4, D64.8, D68- D68.6, D68.8- D68.9, D69- D69.4, D69.6, D69.8, D70- D70.4, D70.8- D70.9, D72- D72.1, D72.8- D72.9, D73- D73.5, D73.8- D73.9, D74.0, D74.8- D74.9, D75- D75.2,D75.8- D75.9, D76- D76.3, D80- D80.9, D81- D81.9, D82- D82.4, D82.8- D82.9, D83- D83.2, D83.8- D83.9,D84- D84.1, D84.8- D84.9, D86.8, D89- D89.2, D89.8- D89.9, |
| **E codes group** |
| E03- E03.1, E03.3- E03.5, E03.8- E03.9, E04- E04.2, E04.8- E04.9, E05- E05.5, E05.8- E05.9, E06- E06.3, E06.5, E06.9, E07- E07.1, E07.8- E07.9, E16.1- E16.4, E16.8- E16.9, E20- E20.1, E20.8- E20.9, E21- E21.5, E22- E22.2, E22.8- E22.9, E23.0, E23.2- E23.3,E23.6- E23.7, E24- E24.1, E24.3, E24.9, E25.0, E25.8- E25.9, E26- E26.1, E26.8- E26.9, E27- E27.2, E27.4- E27.5, E27.8- E27.9, E28- E28.1, E28.3, E28.8- E28.9, E29- E29.1, E29.8- E29.9, E30- E30.1, E30.8- E30.9, E31- E31.2, E31.8- E31.9, E32- E32.1, E32.8- E32.9, E34- E34.5, E34.8- E34.9, E67- E67.3, E67.8, E70- E70.5, E70.8- E70.9, E71- E71.5, E72- E72.5, E72.8- E72.9, E73- E73.1, E73.8- E73.9, E74- E74.4, E74.8- E74.9, E75- E75.6, E76- E76.3, E76.8- E76.9, E77- E77.1, E77.8- E77.9, E79- E79.2, E79.8- E79.9, E80- E80.7, E83- E83.9, E84- E84.9, E85- E85.9, E88- E88.9 |

**Table S2.** Novel Inborn Errors of Immunity identified by studying patients from the Middle East and North Africa region.

| | Disease | Gene | Origin in MENA | Parental Consanguinity | Year of publication | Ref. |
|---|---|---|---|---|---|---|
| **1.** | **Immunodeficiencies affecting cellular and humoral immunity** | | | | | |
| **1.** | CD45 deficiency | *PTPRC* | Turkey | 1 | 1997 | PMID: 9068311 |
| **2.** | LAT deficiency | *LAT* | Arab | 1 | 2016 | PMID: 27242165 |
| **3.** | DNA PKcs deficiency | *PRKDC* | Turkey | 1 | 2009 | PMID: 19075392 |
| **4.** | Cernunnos/XLF deficiency | *NHEJ1* | Turkey | 1 | 2006 | PMID:16439204 |
| **5.** | CD40 deficiency | *CD40* | Turkey | 1 | 2007 | PMID:17502893 |
| **6.** | MHC class I deficiency | *TAP2* | Morocco | 1 | 1994 | PMID:7517574 |
| **7.** | MHC class II deficiency group A B C D | *CIITA* | North African | 1 | 2001 | PMID:11313409 |
| **8.** | DOCK8 deficiency | *DOCK8* | Turkey | 1 | 2014 | PMID:14722525 |
| **9.** | DOCK2 deficiency | *DOCK2* | Lebanon / Turkey/ Kuwait | 1 | 2015 | PMID:26083206 |
| **10.** | STK4 deficiency | *STK4* | Turkey | 1 | 2012 | PMID:22174160 |
| **11.** | TCRα deficiency | *TRAC* | Pakistan | 1 | 2011 | PMID: 21206088 |
| **12.** | ITK deficiency | *ITK* | Arab | 1 | 2011 | PMID: 21109689 |
| **13.** | MALT1 deficiency | *MALT1* | Lebanon | 1 | 2013 | PMID: 23727036 |
| **14.** | IL-21 deficiency | *IL21* | Turkey | 1 | 2014 | PMID:24746753 |
| **15.** | IL-21R deficiency | *IL21R* | Lebanon | 1 | 2013 | PMID:24746753 |
| **16.** | OX40 deficiency | *TNFRSF4* | Turkey | 1 | 2013 | PMID: 23897980 |
| **17.** | TFRC deficiency | *TFRC* | Kuwait | 1 | 2016 | PMID: 26642240 |
| **18.** | c-Rel deficiency | *REL* | Kuwait | 1 | 2019 | PMID: 31103457 |
| **19.** | FCHO1 deficiency | *FCHO1* | Turkey /Algeria | *unknown* | 2019 | PMID: 30822429 |
| **20.** | PAX1 deficiency | *PAX1* | Morocco/ Saudi Arabia | 1 | 2020 | PMID: 32111619 |
| **2.** | **Combined immunodeficiencies with associated or syndromic features** | | | | | |
| **21.** | WIP deficiency | *WIPF1* | Morocco | 1 | 2012 | PMID: 22231303 |
| **22.** | Ataxia-telangiectasia | *ATM* | Arab | *unknown* | 1992 | PMID: 1551665 |
| **23.** | Immunodeficiency with centromeric instability and facial anomalies (ICF 3) | *CDCA7* | Turkey | *1* | 2015 | PMID:26216346 |
| **24.** | Immunodeficiency with centromeric instability and facial anomalies (ICF types4) | *HELLS* | Turkey | 1 | 2015 | PMID:26216346 |
| **25.** | POLE2 (Polymerase ε subunit 2) deficiency | *POLE2* | Saudi Arabia | 1 | 2017 | PMID: 26365386 |
| **26.** | ERCC6L2 (Hebo deficiency) | *ERCC6L2* | Pakistan | 1 | 2014 | PMID:24507776 |
| **27.** | MYSM1 deficiency | *MYSM1* | Saudi Arabia | 1 | 2013 | PMID: 24288411 |
| **28.** | MOPD1 deficiency (Roifman syndrome) | *RNU4ATAC* | Lebanon | 0 | 2011 | PMID: 21977988 |
| **29.** | ZNF341 deficiency AR-HIES | *ZNF341* | Arab | 1 | 2018 | PMID: 29907690 |
| **30.** | Comel-Netherton syndrome | *SPINK5* | Pakistan/ Turkey | 1 | 2002 | PMID: 11841556 |
| **31.** | PGM3 deficiency | *PGM3* | Egypt | 1 | 2014 | PMID: 24589341 |
| **32.** | Transcobalamin 2 deficiency | *TCN2* | Lebanon/ Turkey | 1 | 2009 | PMID: 19373259 |
| **33.** | SLC46A1/PCFT deficiency causing hereditary folate malabsorption | *SLC46A1* | Tunisia | *unknown* | 2001 | PMID: 11804211 |
| **34.** | Tricho-Hepato-Enteric Syndrome (THES) | *TTC37* | Pakistan /Kurdish | 1 | 2010 | PMID: 20176027 |
| **35.** | Tricho-Hepato-Enteric Syndrome (THES) | *SKIV2L* | North Africa / Turkey | 1 | 2012 | PMID: 22444670 |
| **36.** | Hepatic veno-occlusive disease with immunodeficiency (VODI) | *SP110* | Lebanon | 1 | 2006 | PMID: 16648851 |
| **37.** | EPG5 deficiency (Vici syndrome) | *EPG5* | Arab / Turkey | 1 | 2013 | PMID: 23222957 |
| **38.** | HOIP deficiency | *RNF31* | Kuwait | 1 | 2015 | PMID: 26008899 |
| **39.** | Hennekam-lymphangiectasia-lymphedema syndrome | *CCBE1* | Oman/ Iraq | 1 | 2009 | PMID:19935664 |
| **40.** | Activating de novo mutations in nuclear factor erythroid 2- like (NFE2L2) | *NFE2L2* | Qatar | 0 | 2017 | PMID: 29018201 |
| **41.** | IL6ST deficiency | *IL6ST-AR* | Saudi Arabia | 1 | 2019 | PMID: 31130284 |
| **3.** | **Predominantly antibody deficiencies** | | | | | |
| **42.** | μ heavy chain deficiency | *IGHM* | Turkey | 1 | 1996 | PMID: 8890099 |
| **43.** | Igα deficiency | *CD79A* | Turkey | 0 | 1999 | PMID: 10525050 |
| **44.** | CD19 deficiency | *CD19* | Turkey | 1 | 2006 | PMID: 16672701 |
| **45.** | CD81 deficiency | *CD81* | Morocco | 1 | 2010 | PMID: 20237408 |
| **46.** | CD20 deficiency | *CD20* | Turkey | 1 | 2010 | PMID: 20038800 |
| **47.** | TRNT1 deficiency | *TRNT1* | Pakistan | 1 | 2014 | PMID: 25193871 |
| **48.** | ATP6AP1 deficiency | *ATP6AP1* | Tunisia | 1 | 2016 | PMID: 27231034 |
| **49.** | AID deficiency | *AICDA* | Morocco / Turkey | 1 | 2000 | PMID: 11007475 |
| **50.** | FNIP1 deficiency | *FNIP1* | Turkey/ Kurdish | 1 | 2020 | PMID: 32181500 |
| **4.** | **Diseases of immune dysregulation** | | | | | |
| **51.** | UNC13D/Munc13–4 deficiency (FHL3) | *UNC13D* | Morocco | 1 | 2003 | PMID: 14622600 |

| | | | | | | |
|---|---|---|---|---|---|---|
| **52.** | Syntaxin 11 deficiency (FHL4) | *STX11* | Kurdish | 1 | 2005 | PMID: 15703195 |
| **53.** | STXBP2/Munc18–2 deficiency (FHL5) | *STXBP2* | Saudi Ara/ Turkey | 1 | 2009 | PMID: 19804848 |
| **54.** | Chediak-Higashi syndrome | *LYST* | Kuwait/ Turkey | 1 | 1997 | PMID: 9215679 |
| **55.** | Griscelli syndrome type 2 | *RAB27A* | Turkey | 1 | 2000 | PMID: 10835631 |
| **56.** | Hermansky-Pudlak syndrome type 10 | *AP3D1* | Turkey | 1 | 2016 | PMID: 26744459 |
| **57.** | LRBA deficiency | *LRBA* | Arab / Iran | 1 | 2012 | PMID: 22608502 |
| **58.** | DEF6 deficiency | *DEF6* | Pakistan/ Iraq | 1 | 2019 | PMID: 31308374 |
| **59.** | FERMT1 deficiency | *FERMT1* | North African | 1 | 2003 | PMID: 12668616 |
| **60.** | Prolidase deficiency | *PEPD* | Middle east | 0 | 1990 | PMID: 2365824 |
| **61.** | IL-10R deficiency | *IL10RA* | Lebanon | 1 | 2009 | PMID: 19890111 |
| **62.** | TGFB1 deficiency | *TGFB1* | Pakistan | 1 | 2018 | PMID: 29483653 |
| **63.** | RIPK1 | *RIPK1* | Pakistan | 1 | 2018 | PMID: 30026316 |
| **64.** | FADD deficiency | *FADD* | Pakistan | 1 | 2010 | PMID: 21109225 |
| **65.** | CD27 deficiency | *CD27* | Morocco | 1 | 2012 | PMID: 22197273 |
| **66.** | CD70 deficiency | *CD70* | Egypt/ Turkey/ Iran | 1 | 2017 | PMID:28011863 |
| **67.** | RASGRP1 deficiency | *RASGRP1* | Turkey | 1 | 2016 | PMID: 27776107 |
| **68.** | RLTPR deficiency | *CARMIL2* | Morocco/ Tunisia/ Turkey | 1 | 2016 | PMID: 27647349 |
| **69.** | PRKCD deficiency | *PRKCD* | Turkey | 1 | 2013 | PubMed: 23319571 |
| **5.** | **Congenital defects of phagocyte number or function** | | | | | |
| **70.** | HAX1 deficiency (Kostmann Disease) (SCN3) | *HAX1* | Kurdish / Turkey/ Iran/ Lebanon | 1 | 2007 | PMID: 17187068 |
| **71.** | G6PC3 deficiency (SCN4) | *G6PC3* | Turkey | 1 | 2009 | PMID: 19118303 |
| **72.** | VPS45 deficiency (SCN5) | *VPS45* | Arab | 1 | 2013 | PMID: 23738510 |
| **73.** | JAGN1 deficiency | *JAGN1* | Algeria/ Iran/ Turkey Morocco/ Pakistan | 1 | 2014 | PMID: 25129144 |
| **74.** | 3-Methylglutaconic aciduria | *CLPB* | Turkey | 0 | 2015 | PMID: 25597510<br>PMID: 25597511<br>PMID: 25650066 |
| **75.** | SMARCD2 deficiency | *SMARCD2* | Pakistan/ Lebanon | 1 | 2017 | PMID: 28369036 |
| **76.** | Shwachman-Diamond Syndrome | *DNAJC21* | Algeria /Pakistan | 1 | 2016 | PMID: 12496757 |
| **77.** | Shwachman-Diamond Syndrome | *EFL1* | Arab | 1 | 2017 | PMID: 28331068 |
| **78.** | Leukocyte adhesion deficiency type 2 (LAD2) | *SLC35C1* | Turkey /Arab | *unknown* | 2001 | PMID: 11326279 |
| **79.** | Leukocyte adhesion deficiency type 3 (LAD3) | *FERMT3* | Turkey | 1 | 2007 | PMID: 17185466 |
| **80.** | Papillon-Lefèvre syndrome | *CTSC* | Egypt / Pakistan/ Lebanon | 1 | 1999 | PMID: 10581027 |
| **81.** | WDR1 deficiency | *WDR1* | Qatar | 1 | 2016 | PMID: 27557945 |
| **82.** | Autosomal recessive CGD | *CYBC1* | Saudi Arabia | 1 | 2017 | PMID: 28600779 |
| **6.** | **Defects in intrinsic and innate immunity** | | | | | |
| **83.** | IL-12 and IL-23 receptor β1 chain deficiency | *IL12RB1* | Turkey | 1 | 1998 | PMID: 9603733 |
| **84.** | IL-12p40 (IL-12 and IL-23) deficiency | *IL12B* | Pakistan | 1 | 1998 | PMID: 9854038 |
| **85.** | ISG15 deficiency | *ISG15* | Turkey /Iran | 1 | 2012 | PMID: 22859821 |
| **86.** | RORγt deficiency | *RORC* | Saudi Arabia | 1 | 2015 | PMID: 26160376 |
| **87.** | EVER1 deficiency | *TMC6* | Algeria | 1 | 2002 | PMID: 12426567 |
| **88.** | EVER2 deficiency | *TMC8* | Algeria | 1 | 2002 | PMID: 12426567 |
| **89.** | CIB1 deficiency | *CIB1* | Iran | 1 | 2018 | PMID: 30068544 |
| **90.** | IRF9 deficiency | *IRF9* | Algeria | 1 | 2018 | PMID: 30143481 |
| **91.** | IFNAR1 deficiency | *IFNAR1* | Iran | 1 | 2019 | PMID: 31270247 |
| **92.** | TRIF deficiency | *TICAM1* | Saudi Arabia | 1 | 2011 | PMID: 22105173 |
| **93.** | DBR1 deficiency | *DBR1* | Arab | 1 | 2018 | PMID: 29474921 |
| **94.** | CARD9 deficiency | *CARD9* | Iran | 1 | 2009 | PMID: 19864672 |
| **95.** | IL-17RC deficiency | *IL17RC* | Turkey | 1 | 2015 | PMID: 25918342 |
| **96.** | IRAK4 deficiency | *IRAK4* | Saudi Arabia | 1 | 2003 | PMID: 12637671 |
| **97.** | MyD88 deficiency | *MYD88* | Turkey | 1 | 2008 | PMID: 18669862 |
| **98.** | Osteopetrosis | *TNFSF11* | Tunisia/ Kurdish | 1 | 2007 | PMID: 17632511 |
| **99.** | Osteopetrosis | *SNX10* | Arab | 1 | 2012 | PMID: 22499339 |
| **100.** | Osteopetrosis | *TCIRG1* | Turkey | 1 | 2000 | PMID: 10942435 |
| **101.** | IL-18BP deficiency | *IL18BP* | Algeria | 1 | 2019 | PMID: 31213488 |
| **102.** | IFNγ deficiency | *IFNG* | Kuwait | 1 | 2020 | PMID: 32163377 |
| **103.** | TBX21 deficiency | *TBX21* | Morocco/ Qatar | 1 | 2020 | PMID: 33296702 |
| **104.** | NOS2 deficiency | *NOS2* | Iran | 1 | 2020 | PMID: 31995689 |
| **105.** | SNORA31 deficiency | *SNORA31* | Morocco/ Saudi Arabia | 1 | 2019 | PMID: 31806906 |

| | | | | | | |
|---|---|---|---|---|---|---|
| **7.** | **Autoinflammatory disorders** | | | | | |
| **106.** | STING-associated vasculopathy infantile-onset (SAVI) | *TMEM173* | Turkey | *unknown* | 2014 | PMID: 25029335 |
| **107.** | TREX1 deficiency Aicardi-Goutieres syndrome 1(AGS1) | *TREX1* | Pakistan/ Turkey | 1 | 2006 | PMID: 16845398 |
| **108.** | RNASEH2B deficiency AGS2 | *RNASEH2B* | Morocco/ Algeria/ Tunisia | 1 | 2006 | PMID: 16845400 |
| **109.** | RNASEH2C deficiency AGS3 | *RNASEH2C* | Pakistan | 1 | 2006 | PMID: 16845400 |
| **110.** | SAMHD1 deficiency AGS5 | *SAMHD1* | Pakistan /Morocco/ Arab | 1 | 2009 | PMID: 19525956 |
| **111.** | Pediatric systemic lupus erythematosus due to DNASE1L3 deficiency | *DNASE1L3* | Arab | 1 | 2011 | PMID: 22019780 |
| **112.** | Spondyloenchondro-dysplasia with immune dysregulation(SPENCD) | *ACP5* | Turkey/ Pakistan / Egypt | 1 | 2011 | PMID: 21217755 |
| **113.** | USP18 deficiency | *USP18* | Turkey | 1 | 2016 | PMID: 27325888 |
| **114.** | Familial Mediterranean fever | *MEFV* | Iraq /North African | *unknown* | 1997 | PMID: 9288758 |
| **115.** | NLRP1 deficiency | *NLRP1* | Algeria | 1 | 2016 | PMID: 27965258 |
| **116.** | ADAM17 deficiency | *ADAM17* | Lebanon | 1 | 2011 | PMID: 22010916 |
| **117.** | Chronic recurrent multifocal osteomyelitis and congenital dyserythropoietic anemia (Majeed syndrome) | *LPIN2* | Jordan | 1 | 2005 | PMID: 15994876 |
| **118.** | DIRA (Deficiency of the Interleukin 1 Receptor Antagonist) | *IL1RN* | Lebanon | 1 | 2009 | PMID: 19494218 |
| **119.** | DITRA (Deficiency of IL-36 receptor antagonist) | *IL36RN* | Tunisia | 1 | 2011 | PMID: 21848462 |
| **120.** | SLC29A3 mutation | *SLC29A3* | Arab | 1 | 2008 | PMID: 18940313 |
| **121.** | Otulipenia/ORAS | *OTULIN* | Pakistan | 1 | 2016 | PMID: 27523608 |
| **122.** | A20 deficiency | *TNFAIP3* | Turkey | *unknown* | 2016 | PMID: 26642243 |
| **123.** | T cell lymphoma subcutaneous panniculitis-like (TIM3 deficiency) | *HAVCR2* | North African | *unknown* | 2018 | PMID: 30374066 |
| **124.** | STAT2 gain of function | *STAT2* | Morocco | 1 | 2020 | PMID: 32092142 |
| **125.** | HEM1 deficiency | *NCKAP1L* | Iran /UAE/ Saudi Arabia | 1 | 2020 | PMID: 32647003/ PMID: 32646852/ PMID: 32766723 |
| **8.** | **Complement deficiencies** | | | | | |
| **126.** | C1q deficiency due to defects | *C1QA* | Iraq / Turkey / Sudan | 1 | 2011 | PMID: 21654842 |
| **127.** | C1q deficiency due to defects | *C1QB* | Morocco | 1 | 2011 | PMID: 21654842 |
| **128.** | C1q deficiency due to defects | *C1QC* | Saudi Arabia/ Pakistan/ Turkey | 1 | 2011 | PMID: 21654842 |
| **129.** | Membrane Cofactor Protein (CD46) deficiency | *CD46* | Turkey | 1 | 2003 | PMID: 14566051 |
| **130.** | CD55 deficiency (CHAPEL disease) | *CD55* | Turkey /Morocco /Syria / Arab | 1 | 2017 | PMID: 28657829 PMID: 28657861 |
| **9.** | **Bone marrow failure** | | | | | |
| **131.** | Fanconi anemia type E | *FANCE* | Turkey | *unknown* | 2000 | PMID: 11001585 |
| **132.** | Fanconi anemia type G | *XRCC9* | Arab | 1 | 1998 | PMID: 9806548 |
| **133.** | Fanconi anemia type I | *FANCI* | Turkey | 1 | 2007 | PMID: 17452773 |
| **134.** | Fanconi anemia type N | *PALB2* | Morocco | *unknown* | 2007 | PMID: 17200671 |
| **135.** | Fanconi anemia type O | *RAD51C* | Pakistan | 1 | 2010 | PMID: 20400963 |
| **136.** | Fanconi anemia type U | *XRCC2* | Saudi Arabia | 1 | 2012 | PMID: 22232082 |
| **137.** | Dyskeratosis congenita, DKCB1 | *NOLA3* | Saudi Arabia | 1 | 2007 | PMID: 17507419 |
| **138.** | Dyskeratosis congenita, DKCB2 | *NOLA2* | Turkey | 1 | 2008 | PMID: 18523010 |
| **139.** | Dyskeratosis congenita, DKCB4 | *TERT* | Iran/ Libya | 1 | 2007 | PMID: 17785587 |
| **140.** | Dyskeratosis congenita, DKCB6 | *PARN* | Pakistan | 1 | 2015 | PMID: 25893599 |
| **141.** | Coats plus syndrome | *STN1* | Arab | 1 | 2016 | PMID: 27432940 |
| **142.** | Coats plus syndrome | *CTC1* | Egypt | 1 | 2012 | PMID: 22267198 |

*0: non-consanguineous marriage, 1: consanguineous marriage*

**Table S3-** Comparison of Inborn Errors of Immunity epidemiological indexes among different regional and continental registers.

| Reigon/ Country | Population at year of report | Registered Patients | Year of report | PAD (%) | CID (%) | Phagocytic defects (%) | Complement deficiency (%) | Other PIDs (%) | Confirmed genetic diagnosis (%) | Gender ratio M/F | Consanguinity (%) |
|---|---|---|---|---|---|---|---|---|---|---|---|
| Total MENA (Current study) | 578,000,000 | 17120 | 2020 | 41.2 | 15.4 | 11.0 | 1.6 | 30.8 | 4873 (28.4) | 1.5 | 60.5 |
| Total Asia [2] | 464,452067 | 15939 | 2020 | 41.9 | 20.4 | 13.8 | 3.5 | 20.3 | 3161 (25.9) | 2.2 | 20.8 |
| Total Oceania [2] | 42669777 | 1876 | 2020 | 77.4 | 8.9 | 3.2 | 5.9 | 4.6 | 223 (11.8) | 1.1 | NR |
| Total Africa [2] | 1339849038 | 4509 | 2020 | 22.0 | 27.4 | 17.7 | 1.6 | 30.9 | 250 (5.5) | 1.3 | 54.9 |
| Total Europe [2] | 747632000 | 40223 | 2020 | 53.9 | 11.3 | 9.0 | 13.0 | 12.6 | 6239 (15.5) | 0.7 | 3.4 |
| Total America [2] | 1001000000 | 42067 | 2020 | 56.0 | 5.2 | 5.0 | 2.3 | 31.3 | 3979 (9.4) | 2.5 | 0.5 |
| Total World [2] | 7800000000 | 104614 | 2020 | 51.9 | 10.8 | 8.3 | 6.6 | 22.1 | 13852 (13.2) | 1.5 | 6.2 |
